# First recorded food-borne outbreak of gastroenteritis caused by enteroinvasive *Escherichia coli* serotype O8:H19 in Thailand

**DOI:** 10.1007/s10096-024-05024-1

**Published:** 2024-12-24

**Authors:** Kazuhisa Okada, Amonrattana Roobthaisong, Atchareeya Nakkarach, Suthida Muangnoicharoen Hearn, Adirek Saenharn, Lalada Naksen, Pawinee Doung-Ngern, Pilailuk Akkapaiboon Okada, Tetsuya Iida

**Affiliations:** 1 Thailand-Japan Research Collaboration Center on Emerging and Re-emerging Infections, Muang, Nonthaburi, Thailand; 2 Research Institute for Microbial Diseases, Osaka University, Suita, Osaka Japan; 3 Division of Epidemiology, Department of Disease Control, Ministry of Public Health, Muang, Nonthaburi, Thailand; 4 Loei Provincial Health Office, Muang, Loei, Thailand; 5 Department of Medical Sciences, National Institute of Health, Ministry of Public Health, Muang, Nonthaburi, Thailand

**Keywords:** Gastroenteritis, enteroinvasive *Escherichia coli*, *Escherichia coli* O8:H19, food-borne outbreak, *ipaH* gene, whole-genome sequencing

## Abstract

An enteroinvasive *Escherichia coli* (EIEC) outbreak affecting 154 individuals was identified among attendees at a wedding in Loei Province, Thailand. The median time to symptom onset was 18 h (range: 7–72 h). The epidemiological evidence suggested that *larb-neua-dib (*spicy minced raw beef salad) was the probable source of the outbreak. The O8:H19 isolates identified in this study closely resemble the O8:H19 strains from the United States and the United Kingdom, but not the O96:H19 strains from Europe. This is the first EIEC outbreak documented in Thailand. Complexities in identifying EIEC contribute to its underreporting.

**Supplementary Information:**

The online version contains supplementary material available at 10.1007/s10096-024-05024-1.

## Introduction

Enteroinvasive *Escherichia coli* (EIEC) and *Shigella* spp. are closely related bacterial species that share similar virulence mechanisms and disease symptoms [1]. Distinguishing between them is challenging owing to their close genetic relationship, but this is essential for epidemiological and surveillance purposes [2]. Although *Shigella* is associated with large food-borne disease outbreaks, reports of EIEC outbreaks are rare [3]. Because standard biochemical tests cannot distinguish EIEC from commensal *E. coli*, genetic testing is required to identify EIEC strains.

## Outbreak investigation

A group of people who had attended a rural wedding event on 11 March, 2023, presented to Na Duang Hospital in Loei in northeast Thailand with gastroenteritis. On 13 March, 2023, the epidemiological officer at Na Duang Hospital notified the Communicable Disease Control Group at the Loei Provincial Health Office of the outbreak. Initially, 52 cases were identified, of which 14 patients were admitted to the hospital and 38 were identified in the community. Standard culture methods did not result in the identification of a bacterial pathogen; however, 13 of 18 stool specimens showed an *Entamoeba histolytica* morphology based on microscopy, leading to the initial suspicion that *E. histolytica* was the cause of the outbreak.

The Joint Investigation Team from the Loei Provincial Health Office, in collaboration with the Surveillance and Rapid Response Team in Na Duang District, conducted an outbreak investigation. Approximately 500 individuals had attended the wedding, of whom 272 were traced. The case definition for this surveillance included having loose or watery stools at least three times within 24 h, or experiencing at least two of the following symptoms: fever, headache, abdominal pain, nausea, vomiting, or fatigue. Of the attendees traced, 154 met the case definition, three were unwell but did not meet the case definition, and the remaining 115 were asymptomatic. Between 14 and 16 March, stool specimens were collected from 59 attendees who met the case definition and four asymptomatic attendees (Fig. S1).

Stool specimens were analysed upon arrival at the Research Collaboration Center on Emerging and Re-emerging Infections laboratory, located in the suburbs of Bangkok. Multiplex quantitative PCR assays targeting 19 enteropathogens [4] detected the presence of *Shigella* or EIEC (*ipaH* gene) in the first six specimens tested, whereas no parasites, including *E. histolytica*, or viruses were detected. Initial microscopy at the site revealed the presence of cysts in stools with *E. histolytica*-like morphology. However, subsequent genetic testing was negative for *E. histolytica*, suggesting that the cysts may have been *E. dispar*, a morphologically similar but non-pathogenic species. A previous case-control study conducted in eight hospitals in Thailand from April 2016 to March 2018 did not identify an association between *E. histolytica* and acute diarrhoea in hospitalized patients, whereas *ipaH* positivity was significantly associated with hospitalization with acute diarrhoea [5]. Of the remaining 57 specimens, 43 (39 from symptomatic and four from asymptomatic individuals) tested positive for *ipaH*. *Shigella* isolation was attempted using Xylose Lysine Deoxycholate (XLD) agar selective media; however, even after examining many suspected red colonies using PCR, bacteria carrying the *ipaH* gene, which could possibly indicate *Shigella*, were not detected (detailed methods are described in the Supplementary Material). Therefore, attempts were made to detect and isolate EIEC from lactose-fermenting *E. coli*-like colonies collected from XLD agar plates. The presence of background bacteria such as *Klebsiella* and commensal *E. coli* made isolation difficult. EIEC was isolated from nine of 17 stool specimens by screening approximately 100 *E. coli*-like colonies per specimen. All EIEC isolates tested positive for lactose fermentation, lysine decarboxylation, non-motility, and serotype O8. EIEC, similar to *Shigella*, is generally unable to decarboxylate lysine or ferment lactose; however, this pattern is not universal [6].

**Fig. 1:**
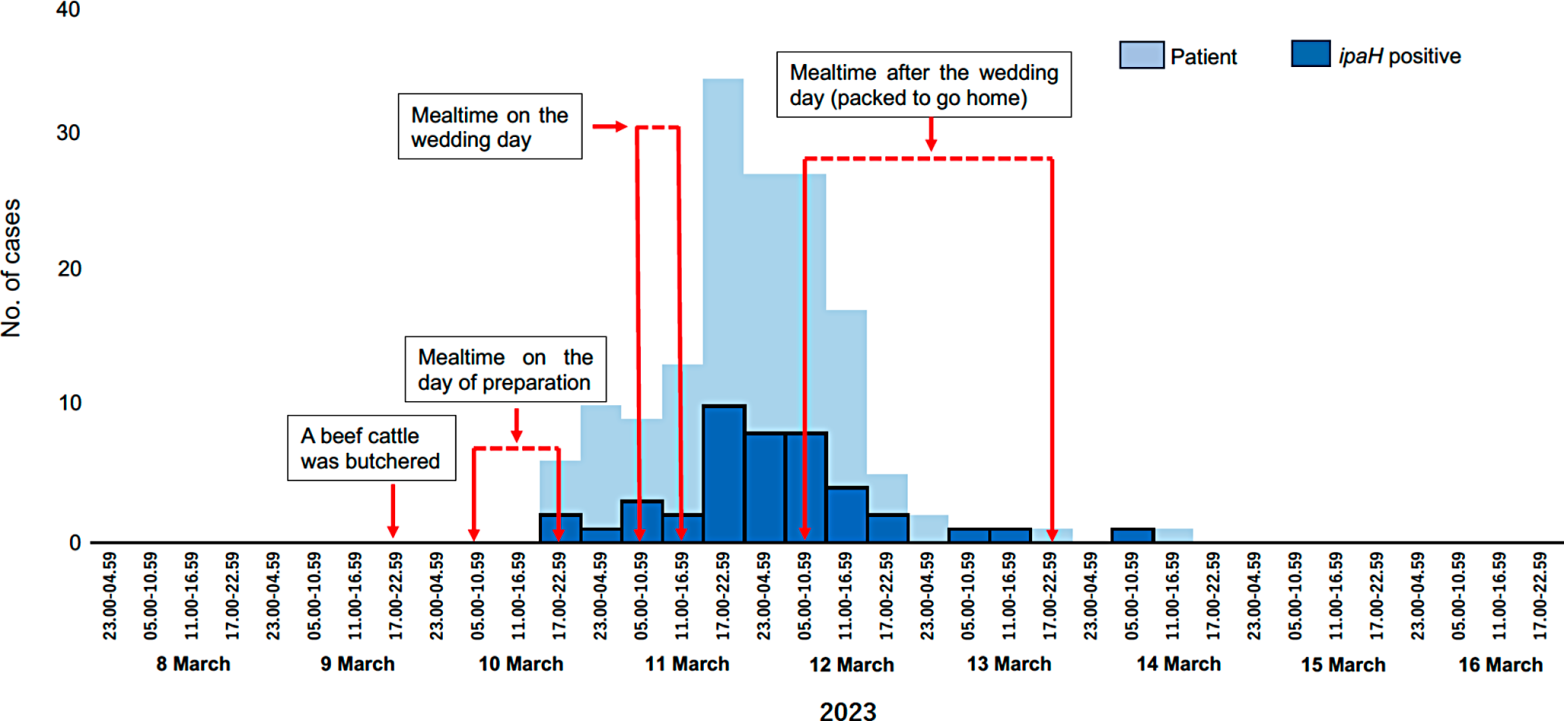
Epidemic curve of 154 patients with the defined illness and *ipaH* gene-positive patients from a wedding event in Na Duang District, Loei Province, Thailand, from 9 to 13 March, 2023

The wedding meal had been prepared and served on 10 March to helpers, including the cooks. The main dish was beef from cattle owned by the host of the wedding that had been butchered on the evening of 9 March (Fig. 1). The wedding meal was served to guests on 11 March. Clinical data were available for 45 patients who tested positive for *ipaH.* The first two cases identified were women, aged 48 and 71 years, of whom one was a cook. These two women had consumed *larb-neua-dib* (spicy minced raw beef salad) at noon on 10 March. They developed symptoms 7 and 8 h later. By 12 March, 11 cooks reported symptoms, and of them, 10 tested positive for *ipaH*. Of the 45 patients, 20 (44%) reported symptom onset on 11 March (the wedding day), and 20 (44%) reported symptom onset on 12 March. The median interval between attending the wedding feast and symptom onset was 18 h (range: 7–72 h). With respect to the eight dishes and four drinks consumed by the 45 patients, 44 (98%) had consumed *larb-neua-dib*, 40 (89%) had consumed ice, and 37 (82%) had consumed water. Other items consumed included beef soup (33/45, 73%) and *yum-ruam-mit* (spicy mixed salad; 15/45, 33%). Of the 45 patients, 24 (53%) were male. The mean age was 52.8 years (range: 5–75 years). Only two patients, aged 5 and 13 years, were under 18 years of age. Leftover raw beef and *larb-neua-dib* from the wedding were sent to the laboratory at the Regional Medical Sciences Center, located near the outbreak site. *Salmonella* spp. group I and *E. coli* were isolated from the raw beef sample, and *E. coli* was isolated from the *larb-neua-dib* sample. These *E. coli* isolates were not available for further testing to determine their similarity to the EIEC isolates. Among the 45 patients, the most common symptoms were watery stools (35/45, 78%) and abdominal pain (35/45, 78%). Other reported symptoms included fever (25/45, 56%), loose stools (19/45, 42%), headache (12/45, 27%), fatigue (10/45, 22%), vomiting (10/45, 22%), nausea (8/45, 18%), mucus stool (7/45, 16%) and bloody stool (2/45, 4%).

**Fig. 2:**
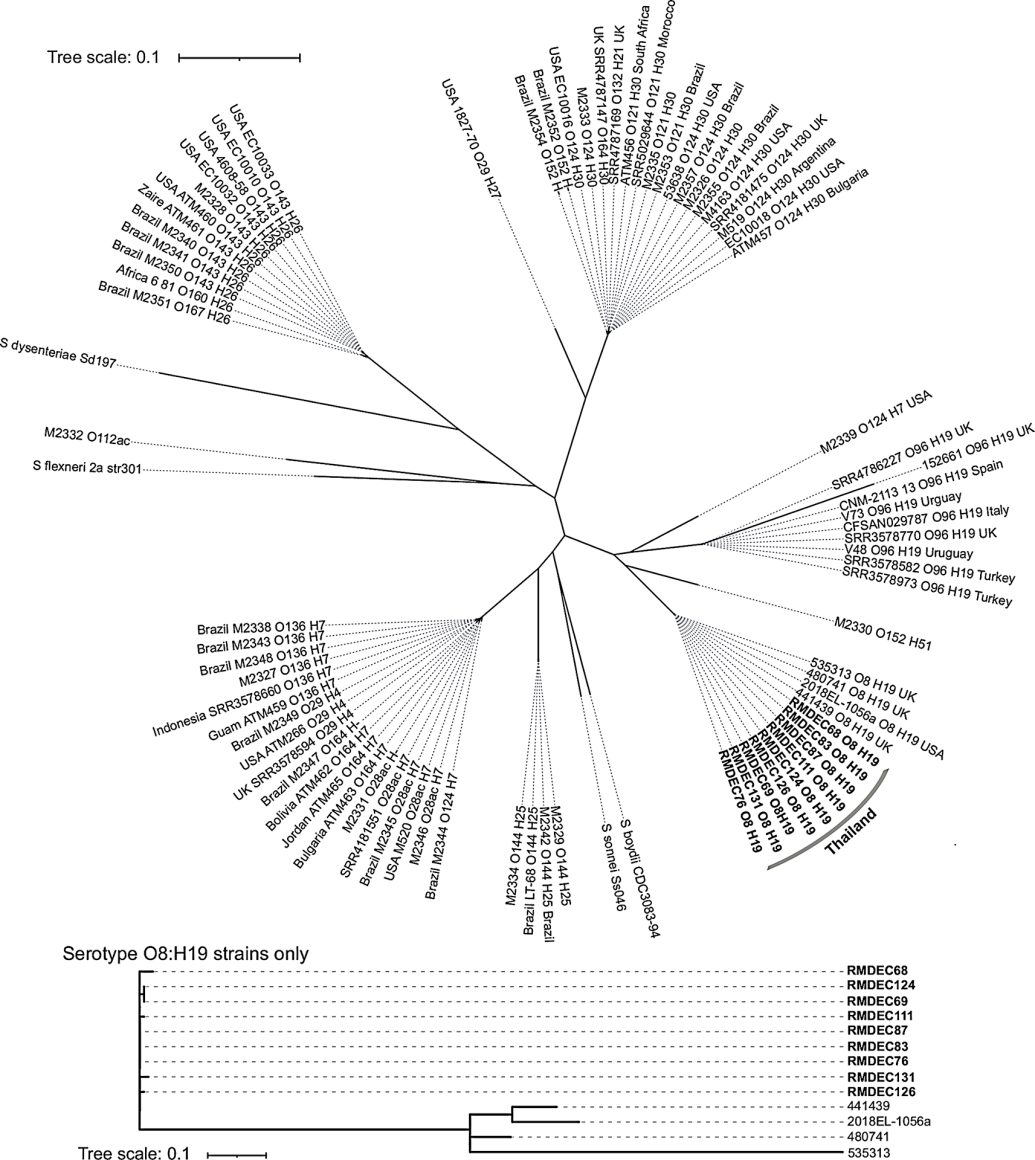
Core genome phylogeny of enteroinvasive *Escherichia coli* (EIEC) outbreak isolates in Thailand and global strains. The figure presents a comparison of the relationship between nine EIEC isolates in Thailand (bold) and other global EIEC strains, alongside four reference *Shigella* species, based on core genome single-nucleotide polymorphisms (SNPs) identified using Parsnp (https://github.com/marbl/parsnp). The visualisation was created using ITOL v.6 (https://itol.embl.de/)

Whole-genome sequencing revealed that the EIEC isolates were serotype O8:H19, sequence type 4267 (ST4267) (Fig. S2), and shared clonality with a maximum distance of three single-nucleotide polymorphisms (Fig. 2) and virulence plasmids measuring 293.8 kb in size (NCBI BioProject id: PRJNA967489). Novel EIEC clones with serotype O96:H19 emerged in Italy in 2012, and serotype O8:H19 emerged in the USA in 2018, and caused outbreaks [7, 8]. Phylogenetic analysis of 80 EIEC strains, representing over 18 serotypes isolated globally [9–11], revealed that the O8:H19 isolates were not closely related to the O96:H19 strains. However, the O8:H19 isolate RMDEC68 shared a virulence plasmid of similar size and features with the O96:H19 strain CFSAN029787 (Fig. S3). Approximately 90% of the genes were common between RMDEC68 and CFSAN029787, and the remaining differences were mostly attributed to insertion elements (transposons) and hypothetical proteins. Additionally, the O8:H19 isolate exhibited a complete set of conjugative elements similar to those found in the O96:H19 strain [9], distinguishing them from other known serotype EIEC strains. The recent emergence of EIEC with transferable virulence plasmids is a matter of concern.

To our knowledge, this is the first report of an EIEC gastroenteritis outbreak in Thailand. The epidemiological evidence suggests that *larb-neua-dib* was the probable source. However, it is unclear whether the EIEC was present in the beef cattle or whether the beef was contaminated by human handling or from other sources, such as water.

EIEC O96:H19 outbreaks in Italy in 2012, the UK in 2014, Sweden in 2017, and Denmark in 2021 were associated with contaminated vegetables, whereas an EIEC O8:H19 outbreak in the USA occurred within an ethnic Nepali refugee community following a potluck gathering with no specific source identified [3, 7, 8, 12, 13]. EIEC is considered to have evolved from commensal *E. coli* via horizontal gene transfer and other genetic changes [9, 10]. The emergent O96:H19 serotype has phenotypic characteristics that resemble those of non-invasive *E. coli* rather than *Shigella*, which could contribute to its improved survival and adaptation to different ecological niches [9, 14].

## Conclusions

The epidemiological evidence suggests that *larb-neua-dib* was the probable source of this EIEC O8:H19 outbreak. The organism was identified through rigorous investigation using a combination of epidemiological, microbiological, and genetic methods. EIEC outbreaks are underreported owing to challenges with identification. An improved understanding of the epidemiology of EIEC could lead to improved prevention and control.

## Electronic Supplementary Material

Below is the link to the electronic supplementary material.

Supplementary Material 1

## Data Availability

Sequence data that support the findings of this study have been deposited in GenBank under accession code PRJNA967489.
