# Supplementary material for: First recorded food-borne outbreak of gastroenteritis caused by enteroinvasive Escherichia coli serotype O8:H19 in Thailand
Source: Eur J Clin Microbiol Infect Dis. 2024 Dec 24;44(3):733–7. doi: 10.1007/s10096-024-05024-1 (PMC11880058; doi:10.1007/s10096-024-05024-1)
Supplement: Supplementary file 1 — Supplementary Material 1 [file 10096_2024_5024_MOESM1_ESM.pdf]

Supplementary Methods

Hospital standard operating procedures

The hospital's standard operating procedure for culturing bacteria from faecal samples to detect *Shigella, Salmonella, Vibrio, Aeromonas*, and *Plesiomonas shigelloides* was as follows: Stool samples were resuspended and streaked onto MacConkey agar, *Salmonella-Shigella* (SS) agar or Xylose Lysine Deoxycholate (XLD) agar, and thiosulfate citrate bile salts sucrose (TCBS) agar. Colonies of suspected pathogens on selective agars were then identified through standard biochemical and serotyping tests.

Isolation and characterisation of enteroinvasive *Escherichia coli*

Faecal specimens that tested positive for *ipaH* gene were plated on XLD agar both directly and after enrichment culture. Both suspected colonies of *Shigella* and *E. coli* were selected. The presence of the *ipaH* gene in colonies was then screened using PCR amplification. A primer set (Shi-F3: 5′-CGCCTTTCCGATACCGTCTC and Shi-B3: 5′-CTGATGGACCAGGAGGGT) [1] and Quick Taq HS DyeMix (Toyobo, Japan) were used for the PCR amplification. The PCR cycling protocol included an initial denaturation at 94 °C for 5 min, followed by 30 cycles of 94 °C for 30 s, 60 °C for 30 s, 72 °C for 30 s, and a final extension at 72 °C for 5 min. The isolates were identified using conventional methods and the VITEK2 identification card system (bioMérieux, France). Serotyping was conducted for *E. coli* using commercially available antisera (Denka Seiken, Japan).

Preparation of RNA and DNA from stool specimens for multiplex quantitative PCR

Total RNA and DNA were extracted from stool samples using QIAamp Viral RNA and QIAamp Fast DNA Stool Mini Kits (Qiagen, USA) on a robotic workstation or manually. To extract viral RNA/DNA, up to 200 mg of stool (or the maximum amount available) was suspended in saline to prepare a 10% stool suspension; however, some specimens did not contain enough stool to achieve this concentration. The supernatant was mixed with buffer AVL containing carrier RNA. Viral genomes were extracted, eluted with buffer AVE,

and stored at −80 °C. For bacterial and parasitic DNA extraction, a stool pellet of up to 200 mg (or the maximum amount available) was obtained by centrifuging at 15,000 × *g* for 1 minute. The pellet was mixed with an appropriate amount of InhibitEX buffer, heated at 70 °C for 5 minutes, and then centrifuged at 18,000 × *g* for 2 minutes. A 200-µL aliquot was used for genomic DNA extraction, which was eluted with 50 µL of buffer and stored at −25 °C. The reactions were prepared in a final volume of 15 µL and contained 1.5 µL template, 0.4 µM forward and reverse primers, 0.2 µM probes, 1× QuantiFast Pathogen master mix, 1× ROX dye solution, and 1× QuantiFast RT mix. The RT mix was included only for viral RNA samples. Exogenous internal positive control reactions were prepared separately and contained 1× QuantiFast Pathogen master mix, 1× ROX dye solution, 1× IC assay mixture, 1× IC template (RNA or DNA), and 1× QuantiFast RT mix (for RNA detection). Further details are provided in the reference by Wongboot et al. [2].

Genome sequencing and genomic characterisation

A single EIEC isolate was selected from each stool sample for whole-genome sequencing (WGS) using the DNeasy Blood & Tissue Kits (Qiagen, Germany), following the manufacturer's instructions. The genome libraries were sequenced on the Illumina MiSeq platform using a reagent kit v3 (600 cycles). DNA library preparation was performed with 500 ng of genomic DNA using the Illumina DNA Prep kit (Illumina, San Diego, CA, USA) and Nextera DNA CD Indexes (96 Indexes; Illumina), according to the manufacturer's instructions. To preprocess the Illumina reads, Fastp (v.0.20.1) was used to detect and remove adapters and bases with a Phred quality score below 30 [3]. The complete genome sequence of the O8:H19 isolate RMDEC68 was analysed using Unicycler (v.0.4.8) [4], which incorporated data from Illumina and Oxford Nanopore MinION sequencing. For ONT reads, sequencing library preparation was conducted using the Native barcoding kit (SQK-NBD114.24), and the resulting data, after adapter trimming, were subsequently filtered using filtlong v.0.2.0 [5].

The sequence reads or assembly sequences were analysed using TORMES v1.3.0, an automated pipeline for whole bacterial genome analysis [6]. Briefly, clean reads were used for *de novo* assembly with SPAdes v3.15.2 [7], and the resulting contig sequences were annotated using Prokka v1.14.6 [8]. Multilocus sequence typing (MLST) was performed with mlst v2.19.0 [9, 10]. Serotyping analysis for *E. coli* was conducted using SerotypeFinder [11]. The 'gff' files generated with Prokka were employed for pangenome comparison between samples using Roary v3.13.0, based on the presence/absence of predicted genes [12]. ShigEiFinder was used to identify 38 virulence genes from the pINV invasive plasmid to determine whether the plasmid is present in the genome [13]. Single-nucleotide polymorphism calling and phylogenetic inference single-nucleotide polymorphisms (SNPs) were identified using Harvest version 1.0.1, which is available at (https://github.com/marbl/harvest) [14].

References

1. Wang Y, Wang Y, Luo L, Liu D, Luo X, Xu Y, Hu S, Niu L, Xu J, Ye C (2015) Rapid and Sensitive Detection of Shigella spp. and Salmonella spp. by Multiple Endonuclease Restriction Real-Time Loop-Mediated Isothermal Amplification Technique. Front Microbiol 6:1400.
2. Wongboot W, Okada K, Chantaroj S, Kamjumphol W, Hamada S (2018) Simultaneous detection and quantification of 19 diarrhea-related pathogens with a quantitative real-time PCR panel assay. J Microbiol Methods 151:76-82. https://doi.org/10.1016/j.mimet.2018.06.006.
3. Chen S, Zhou Y, Chen Y, Gu J (2018) fastp: an ultra-fast all-in-one FASTQ preprocessor. Bioinformatics 34 (17):i884-i890.
4. Wick RR, Judd LM, Gorrie CL, Holt KE (2017) Unicycler: Resolving bacterial genome assemblies from short and long sequencing reads. PLoS Comput Biol 13 (6):e1005595.
5. Wick RR (2021) Filtlong. https://github.com/rrwick/Filtlong.
6. Quijada NM, Rodriguez-Lazaro D, Eiros JM, Hernandez M (2019) TORMES: an automated pipeline for whole bacterial genome analysis. Bioinformatics 35 (21):4207-4212.

7. Prjibelski A, Antipov D, Meleshko D, Lapidus A, Korobeynikov A (2020) Using SPAdes De Novo Assembler. Curr Protoc Bioinformatics 70 (1):e102.
8. Seemann T (2014) Prokka: rapid prokaryotic genome annotation. Bioinformatics 30 (14):2068-2069.
9. Seemann T, mlst. Github https://github.com/tseemann/mlst
10. Jolley, K.A., Maiden, M.C (2010) BIGSdb: Scalable analysis of bacterial genome variation at the population level. BMC Bioinformatics 11:595.
11. Joensen KG, Tetzschner AM, Iguchi A, Aarestrup FM, Scheutz F (2015) Rapid and Easy In Silico Serotyping of Escherichia coli Isolates by Use of Whole-Genome Sequencing Data. J Clin Microbiol 53: 2410–2426.
12. Page AJ, Cummins CA, Hunt M, Wong VK, Reuter S, Holden MT, Fookes M, Falush D, Keane JA, Parkhill J (2015) Roary: rapid large-scale prokaryote pan genome analysis. Bioinformatics 31 (22):3691-3693.
13. Zhang X, Payne M, Nguyen T, Kaur S, Lan R (2021) Cluster-specific gene markers enhance Shigella and enteroinvasive Escherichia coli in silico serotyping. Microb Genom 7(12):000704.
14. Treangen TJ, Ondov BD, Koren S, Phillippy AM (2014) The Harvest suite for rapid core-genome alignment and visualization of thousands of intraspecific microbial genomes. Genome Biol 15 (11):524.

Supplementary Figures

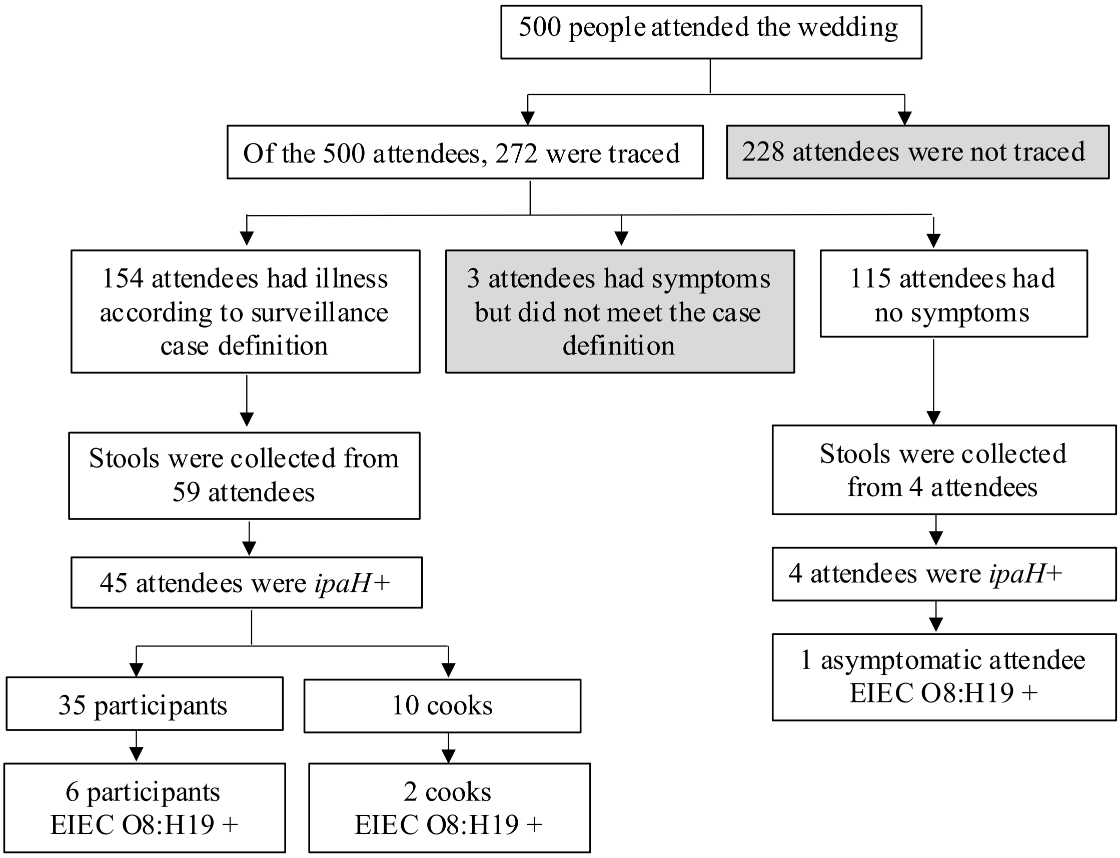

Fig. S1. Flowchart showing the number of individuals involved at each stage of the study.

										Symptoms											Foods on March 10, 2023			Foods on March 11, 2023				Beverages			
**Isolate**	**Serotype**	**Sequence type**	**Gender**	**Age**	**Group**	**Eating date**	**Time**	**Date of onset**	**Time**	Watery stool	Loose stool	Mucus stool	Bloody stool	Abdominal pain	Fever	Nausea	Vomiting	Abdominal cramps	Fatigue	Headache	*Larb-neua-dib*	Beef soup	*Yum-ruam-mit*	*Larb-neua-dib*	Beef soup	*Yum-ruam-mit*	Sticky rice	Soft drink	Water	Ice	Liqour
RMDEC-87	O8:H19	4267	Male	54	Participant	March 10, 2023	9:00	March 11, 2023	3:00	■				■		■					■	■		■	■				■	■	
RMDEC-69	O8:H19	4267	Male	22	Cook	March 10, 2023	18:00	March 11, 2023	18:00	■	■	■		■							■	■		■	■			■		■	■
RMDEC-126	O8:H19	4267	Female	62	Participant	March 10, 2023	19:00	March 11, 2023	6:00	■				■	■		■				■	■		■	■				■	■	
RMDEC-131	O8:H19	4267	Male	72	Participant	March 11, 2023	8:00	March 12, 2023	2:00	■	■	■		■				■	■					■	■		■		■	■	
RMDEC-68	O8:H19	4267	Male	13	Participant	March 11, 2023	9:00	March 14, 2023	9:00	■					■									■	■	■			■	■	
RMDEC-83	O8:H19	4267	Female	59	Participant	March 11, 2023	9:30	March 12, 2023	12:00	■	■													■	■				■	■	
RMDEC-124	O8:H19	4267	Female	25	Cook	March 11, 2023	14:00	March 12, 2023	14:10	■				■	■									■					■	■	
RMDEC-111	O8:H19	4267	Female	50	Participant	March 12, 2023	8:00	March 12, 2023	17:00		■			■		■								■	■				■	■	
RMDEC-76	O8:H19	4267	Male	47	Participant	March 11, 2023	11:30	Asymptomatic case																■							

Fig. S2. Background Information and characteristics of sequenced EIEC isolates

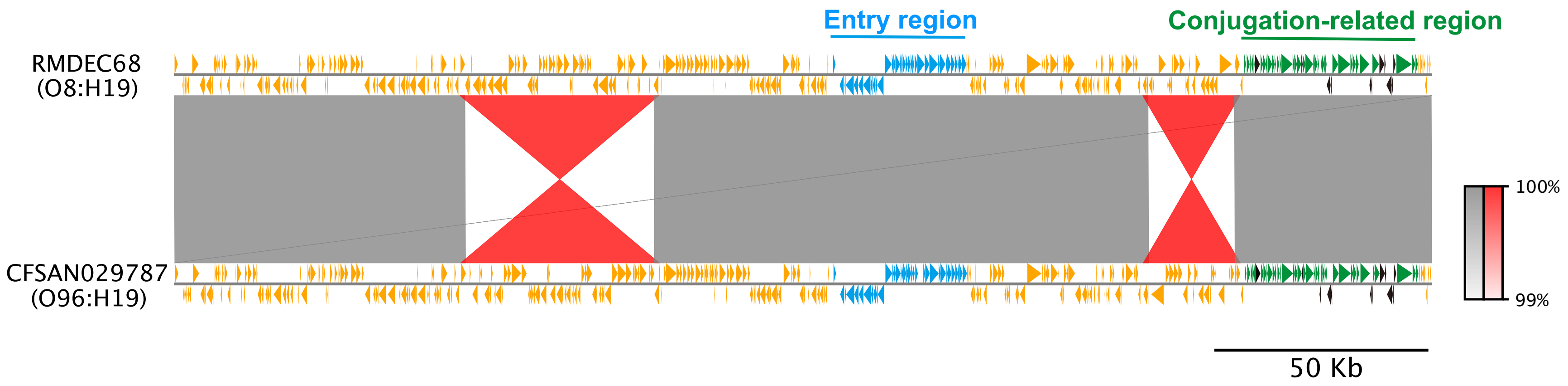

Fig. S3. Comparison of the virulence plasmids in two EIEC isolates using pyGenomeViz with MUMmer alignment. Grey linkages represent nucleotide similarity percentages, while red linkages denote inverted sequence similarities. Arrowheads indicate gene locations and transcriptional direction. Genes are colour-coded as follows: blue for the entry region genes, green for conjugation-related genes, and black for genes unrelated to conjugation.
